# Successful recovery of associated interstitial nephritis and focal segmental glomerulosclerosis in patients with HCV and HIV treated with sofosbuvir and daclatasvir and revision of literature 

**DOI:** 10.5414/CNCS109221

**Published:** 2018-10-26

**Authors:** Michele Mirabella, Lucia Taramasso, Laura Ambra Nicolini, Rodolfo Russo, Claudio Viscoli, Antonio Di Biagio

**Affiliations:** 1 Infectious Diseases Unit,; 2 Nephrology Unit, and; 3 Infectious Diseases Unit, Ospedale Policlinico San Martino, Genoa, Italy

**Keywords:** HIV/HCV coinfection, mycophenolate mofetil, focal segmental glomerulosclerosis, sustained viral response, kidney disease

## Abstract

In this report, we describe the coexistence of two rare and debated complications of hepatitis C virus (HCV) infection: interstitial nephritis, with associated focal glomerulosclerosis, and autoimmune hepatitis, in a 55-year-old HIV/HCV-coinfected woman. The patient was treated for the immune-mediated manifestations with mycophenolate mofetil, which she continued for 9 years, as symptoms relapsed at every attempt to discontinue immunosuppression. The patient finally cleared HCV infection thanks to new direct-acting agents and could discontinue immunosuppressive therapy maintaining stable conditions and laboratory parameters after 24-weeks follow-up.

## Introduction 

Hepatitis C virus (HCV)-infected patients are a population at risk of viral-related liver complications, such as fibrosis, cirrhosis, and hepatocellular carcinoma (HCC) [1]. Moreover, immune-mediated extra-liver complications are also possible [2], and among these, renal complications are frequent [3]. The renal diseases mainly associated with HCV infection are membranoproliferative glomerulonephritis, but also membranous nephropathy, IgA nephropathy, proliferative glomerulonephritis, interstitial nephritis, and focal segmental glomerulosclerosis [4]. In human immunodeficiency virus (HIV)-infected patients, other risks factors for development of renal disease are also present, related to HIV infection itself, but also to some antiretroviral therapies [5, 6]. In patients coinfected with HIV/HCV, the causes of renal damage cumulate, creating a pairing of viral infection and renal disease that is commonly observed, but not inseparable. The availability of new direct-acting antivirals (DAAs) has changed the prognosis of HCV infection, increasing significantly efficacy and safety compared to pegylated interferon (pegIFN)-based treatment [3]. However, data regarding the outcome of renal extrahepatic diseases associated with HCV are still scarce. In this report, we describe the outcome of a severe interstitial nephritis following HCV treatment with DAA in an HIV/HCV-coinfected patient and a review of the inherent literature. 

## Clinical case 

**Figure 1.**
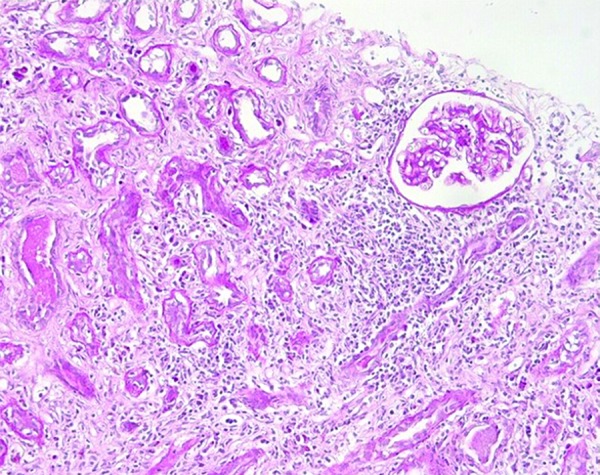
Biopsy specimen with a portion of renal cortical and medulla containing 12 glomeruli, 2 of which were sclerotic. Three glomeruli presented slight segmental mesangial sclerosis, 2 others presented mesangial sclerosis and irregular thickening of blood vessel walls (due to ischemia). Diffuse edema, interstitial fibrosis, focal atrophy, and tubular deterioration were present among with diffuse lymphomonocytic infiltration with infiltration of the tubular tissue. Arteriosclerosis was present. Immunofluorescence tests were negative. Electronic microscopy was not performed. The specimen was compatible with FSGS and interstitial nephritis.

**Figure 2.**
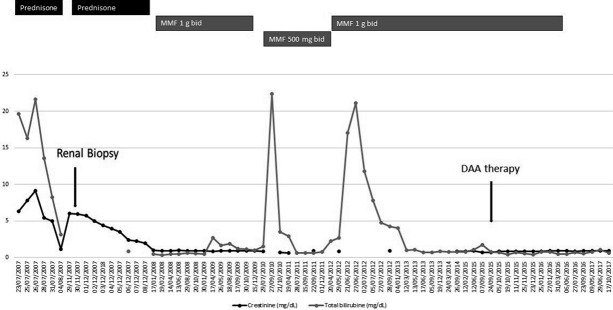
Serial serum creatinine and total bilirubin levels are reported with regard to immunosuppressive and anti-HCV treatment.

A 55-year-old woman, with HIV/HCV (genotype 3a) coinfection diagnosed in 1991, and previous intravenous drug user, has been followed by our outpatient department since 1998. The patient had been treated with antiretrovirals since 1991, with a good immunovirologic response. In 2005, she received anti-HCV therapy with pegIFN and ribavirin for 24 weeks, but the HCV infection relapsed after treatment discontinuation. Hereafter, she regularly followed her antiretroviral therapy and had an HIV-RNA load persistently < 50 copies/mL and stable clinical condition. Despite this, in summer 2007, she developed two consecutive episodes of acute kidney injury (AKI) associated with cholestatic acute hepatitis. At the time of her first hospital admission, her antiretroviral therapy (ART) with tenofovir disoproxil fumarate 245 mg q.d., lamivudine 300 mg q.d., and fosamprenavir 700 mg b.i.d. was stopped, in the suspicion of possible renal damage. Blood tests revealed acute kidney failure, with a serum creatinine level of 6.3 mg/dL associated with blood urea nitrogen 67 mg/dL and uric acid 10.6 mg/dL. Other notable results were hyperbilirubinemia (19.8 mg/dL, normal range 0.20 – 1.20 mg/dL), hepatic failure, with a coagulative deficit (prothrombin time 25), and metabolic acidosis. Anti-smooth-muscle-antibodies (ASMA) were positive (160 : 1), while antinuclear antibodies (ANA), antibodies directed against proteins that bind to nucleic acids (ENA), antibodies directed against phospholipids and antineutrophil cytoplasmic (ANCA), adrenal cortex antibodies (ACA), antimitochondrial antibodies (AMA), and anti-liver-kidney microsomal (ALKM) antibody were negative. Urinalysis showed proteinuria, hematuria, and pyuria. Abdomen ultrasound showed a liver enlargement without focal lesions, a modest ascites, and enlarged kidneys with an increment of arterials resistances. The patient’s management required hemodialysis, albumin, vitamin K, and human plasma. At the same time, the patient started methylprednisolone therapy (250 mg daily, then reduced to 40 mg daily). After clinical and chemistry improvement, the patient was discharged with oral steroid treatment (prednisone 25 mg daily), which was subsequently tapered. Three months later, after steroid discontinuation and ART still discontinued, a new onset of AKI (serum creatinine levels 6.0 mg/dL) required hospital admission. A kidney biopsy was performed showing tubule-interstitial nephritis and focal glomerulosclerosis (Figure 1). Blood tests confirmed the presence of ASMA antibodies as well as negativity for ANA, ENA, p-ANA, c-ANCA, LKM (liver-kidney microsomal), ACA, and AMA. Then, methylprednisolone 40 mg daily was restarted with success (Serial serum creatinine levels are reported in Figure 2). At discharge, oral prednisone (37.5 mg daily) was prescribed, and it was tapered over a period of 4 weeks. In January 2008, considering the progressive improvement of renal function (creatinine 1.0 mg/dL), the consultant nephrologist suggested stopping steroid treatment and starting maintaining treatment with mycophenolate mofetil (MMF), 1,000 mg b.i.d. Following the starting of MMF, the patient exhibited negative ASMA antibodies. 

She also restarted ART with abacavir 300 mg b.i.d., lamivudine 150 mg b.i.d., and lopinavir/ritonavir 400/100 b.i.d.. MMF was continued with good adherence and tolerance until November 2009, when, after 2 years of good clinical condition, it was discontinued. Four weeks after the suspension of MMF, the patient experienced a new deterioration of liver and renal function that required the reintroduction of MMF, at a dosage of 500 mg b.i.d., and new hospital admission (Figure 1). In the following years, she continued ART, switching to darunavir/ritonavir (800/100 mg), etravirine 200 mg × 2, and maraviroc 150 mg × 2 and continued MMF. In June 2012, a new episode of cholestatic hepatitis developed, without renal involvement. A liver biopsy was obtained showing coexisting chronic HCV-related hepatitis and autoimmune hepatitis. Immunosuppressive therapy was enhanced with steroidal therapy (1 mg/kg), but she refused to continue. Thus, she was put on an increased dosage of MMF, of 1,000 mg b.i.d.. This therapy was continued in the following years with improvement of renal and hepatic parameters. In September 2015, the patient received anti-HCV treatment with sofosbuvir 400 mg q.d. plus daclatasvir 60 mg q.d. for 12 weeks without ribavirin. Before the starting of HCV treatment, maraviroc was stopped to avoid expected drug-drug interaction. Sustained virological response (SVR) was achieved, and, 3 months later, in agreement with the nephrologists, the patient halved the MMF dosage, and stopped it after other 12 weeks. Subsequent blood tests showed a permanent negativity of HCV-RNA load, with creatinine and hepatic enzymes persistently within the normal range (creatinine persistently < 0.95 mg/dL, and bilirubin < 1.20 mg/dL) and negative ASMA antibodies 12 months after MMF suspension. Since the patient no longer needed to keep an NRTI-sparing regimen, a single-tablet-regimen with rilpivirine/emtricitabine/tenofovir disoproxil fumarate was reintroduced.[Table Table1]

## Discussion 

In this report, we described the coexistence of two rare and debated complications of HCV infection in an HIV/HCV coinfected woman: interstitial nephritis, with associated focal glomerulosclerosis, and autoimmune hepatitis. Pathogenesis of autoimmune hepatitis is still incompletely understood, but it involves genetic, both immune and nonimmune, and environmental triggering factors. While HCV has long been associated with the induction of autoimmune disorders, it can potentially cause both glomerular and tubulointerstitial diseases, although focal segmental glomerulosclerosis and acute interstitial nephritis (AIN) have been rarely reported [4, 7, 10, 11, 12]. The finding of molecular mimicry by cross-reactivity between epitopes of HCV and liver antigens tends to give weight to the argument that autoimmune hepatitis may be triggered by viral hepatitis [13]. 

As all viruses may potentially trigger autoimmune diseases, the possible role of HIV infection has been evaluated. However, the patient developed the first episode of AKI when she had good immune-virological control of HIV with c-ART. Thus, we excluded the role of HIV infection in the present case. 

Beyond viruses, another possible cause for AIN is drug-induced damage. Indeed, many drugs, such as antibiotics, nonsteroidal anti-inflammatory drugs (NSAIDs), and antiretroviral drugs (e.g., indinavir, abacavir, ritonavir, and atazanavir), have been associated with AIN [8]. For this reason, during the first onset of AKI, the patient discontinued cART, but, she developed a second episode of AKI and she was not taking cART or any other drug associated with the risk of AKI. Additionally, despite it being previously reported in her medical history, the patient had not used recreational drugs for many years before the onset of AKI [9]. Thus, in the present case, HCV is the most probable trigger for autoimmune disease. 

Current guidelines suggest the correction of risk factors in case of secondary disease [14], but in our case, the patient had already stopped intravenous drugs abuse and achieved HIV-RNA suppression with ART when symptoms appeared. Additionally, steroid treatment is recommended as the first-line regimen for autoimmune renal diseases, and it may be withdrawn after a period of clinical stability, as it can lead to remission [15]. When steroids fail or disease relapses, it is possible to switch immunosuppressive therapy to a cytotoxic agent (cyclophosphamide, chlorambucil, or MMF) or calcineurin inhibitors (cyclosporin A or tacrolimus). We chose MMF due to the reduced risk of pharmacological interactions. Immunosuppressive therapy with a cytotoxic agent or a calcineurin inhibitor can be stopped after 1 – 2 years if remission has been maintained [16]. Of note, before HCV eradication, we attempted to stop MMF after 2 years of continuous treatment, but our patient experienced a relapse of renal and liver disease within 4 weeks from suspension. On the other hand, following HCV eradication, MMF discontinuation was safe, and the patient did not experience any deterioration of liver and renal function, and ASMA antibodies remained undetectable until the last available follow-up. 

Despite anecdotal experiences showing a favorable impact of SVR on HCV-associated kidney diseases [11, 16, 17, 18], few cases of long-term responders in interstitial nephritis after HCV infection eradication have been reported so far, and none of them following treatment with DAAs [11, 17, 18]. In summary, our patient developed stable and off-treatment remission of interstitial nephritis following SVR with DAAs. HCV eradication following treatment with DAAs did not negatively impact on autoimmune hepatitis, and also, we shut down the trigger of renal disease. We believe this case provides further insight into whether DAA treatment may impact on the outcome of patients with extra-hepatic liver diseases. 

## Funding 

The authors did not receive any financial support for the completion of the manuscript. 

## Conflict of interest 

No potential conflict of interest relevant to this article was reported. 

**Table 1. Table1:** Patient therapies before, during, and after HCV eradication treatment with direct acting agents (DAA). Antiretroviral drugs are highlighted in bold.

Pre-eradication therapy	During eradication with DAA	After eradication
MMF 1 g b.i.d. Nebivolol 5 mg o.d. **Darunavir 600 mg b.i.d. ** **Ritonavir 100 mg b.i.d. ** **Etravirine 100 mg b.i.d. ** **Maraviroc 150 mg b.i.d.** Lorazepam 2.5 mg o.d. Amitriptyline 60 mg o.d.	MMF 1 g b.i.d. Nebivolol 5mg o.d. **Darunavir 600 mg b.i.d. ** **Ritonavir 100 b.i.d. ** **Etravirine 100 mg b.i.d. ** **Sofosbuvir 400 mg o.d. ** **Daclatasvir 60 mg o.d.** Lorazepam 2.5 mg o.d. Amitriptyline 30 mg o.d.	**RPV/FTC/TDF o.d. ** Atenolol 100 mg o.d.

MMF = mycophenolate mofetil; RPV/FTC/TDF = rilpivirine/emtricitabine/tenofovir disoproxil fumarate; b.i.d. = bis in die; o.d. = once daily.
